# Refrigerator ownership and the nutrition-health trade-off: evidence from Chinese households

**DOI:** 10.3389/fnut.2025.1620134

**Published:** 2025-07-08

**Authors:** Manli Zheng, Rong Cai, Li Zhang, Ke Xu

**Affiliations:** ^1^Institute of Food and Strategic Reserves, Nanjing University of Finance and Economics, Nanjing, China; ^2^School of Economics and Management, Lu’An Vocational Technical College, Lu’An, China; ^3^Collaborative Innovation Center for Modern Grain Circulation and Safety, Nanjing, China

**Keywords:** refrigerator, dietary structure, overweight, obese, the control function approach

## Abstract

**Objective:**

This study investigates the dynamic effects of household refrigerator ownership on dietary patterns and the risk of overweight and obesity. The objective is to offer policy guidance for developing countries that are facing health challenges during their nutritional transition.

**Methods:**

This study utilizes six waves of tracking data from the China Health and Nutrition Survey (CHNS, 1997–2011, *n* = 16,665). To address the estimation bias caused by endogeneity, the Control Function approach is employed. Furthermore, to elucidate the pathways through which refrigerator ownership influences overweight and obesity, a three-stage mediation model is used to assess the mediating effects of food purchases and processed food consumption on these outcomes.

**Results:**

Refrigerator ownership significantly increases daily calorie intake by 39.1%. The ratios of energy derives from fat and protein rose by 0.104 and 0.018 percentage points, respectively, while the carbohydrate energy ratio decreases by 0.12 percentage points, indicating a shift towards a higher energy-dense diet. The mediating mechanism tests identify two pathways: an increased intake of high-fat and high-protein foods due to larger purchases and a rise in the consumption of processed foods. Health effects exhibit considerable heterogeneity; the risk of overweight increases with income, and the impact on older individuals is significantly greater than that on younger groups. Additionally, males face more than double the risk of obesity compared to females.

**Conclusion:**

Refrigerator ownership significantly alters the dietary energy structure and raises the risk of overweight and obesity. These insights hold substantial theoretical and practical value in balancing technological advancements with public health objectives.

## 1 Introduction

Overweight and obesity have become a growing public health issue worldwide. In 2022, nearly 2.5 billion adults were overweight, and over 890 million were obese worldwide (1). These conditions significantly increase the risk of cardiovascular diseases and diabetes, leading to shorter life expectancy and higher healthcare costs (2–4). Global economic losses from overweight and obesity are projected to reach US$3 trillion by 2030, potentially exceeding US$18 trillion by 2060 (5). The rise in overweight and obesity rates is closely linked to the ‘nutrition transition’ experienced by many countries (6). As the most populous developing country, China is undergoing one of the world’s most rapid dietary transformations. According to the National Bureau of Statistics, per capita meat consumption among urban residents increased by 82% from 1990 to 2023, while cereal consumption decreased by 11.6%, and the fat-to-energy ratio continued to rise. While this transition has alleviated malnutrition, it has also created a “double burden of disease,” characterized by excess energy intake and micronutrient deficiencies. The 2020 Report on Nutrition and Chronic Diseases in China shows that over 50% of Chinese adults are overweight or obese, a trend that poses a serious threat to public health and economic development. Studying the dietary structure and obesity trends in China offers valuable insights for balancing health and development worldwide, especially in developing countries.

Among the various factors affecting dietary structure changes, income level is considered a key driving variable. Existing studies have confirmed that income growth significantly improves the nutritional status of low-income groups and effectively promotes a balanced intake of nutrients such as carbohydrates, proteins, and fats (7–9). In addition, whether farmers participate in contract farming and the availability of agricultural markets also affect residents’ diet quality and nutritional health through the mediating effect of income (10, 11). However, this improvement effect diminishes at the margin: when the income level exceeds a certain threshold, the nutritional improvement effect gradually weakens (12). At the same time, rapid urbanization is profoundly reshaping the nutritional health of individuals in developing countries. Urbanization has not only reconfigured food availability and consumption environments through changes in the food supply system—such as the proliferation of fast-food outlets and restaurants (13)—but has also triggered a significant shift in dietary preferences, moving from a traditional high-carbohydrate diet to a modern dietary pattern characterized by high fat and sugar content (14, 15). In the context of digitalization, the widespread adoption of the internet, the use of smartphones, and the popularity of short-video platforms have significantly improved household dietary quality and nutritional intake (16–18). Furthermore, other factors—including the development of free trade, economic growth, cultural shifts, commercialization of agriculture, and the rise of supermarkets—have profoundly influenced the transformation of the population’s dietary patterns (2, 19–22).

Research indicates that poor dietary habits are closely associated with chronic diseases. Specifically, excessive intake of animal protein significantly contributes to the development of these conditions (23). Popkin et al. (24) emphasized a notable positive correlation between nutritional transitions and increasing obesity rates. Overweight and obesity are major factors in the high prevalence of chronic diseases. Notably, the widespread use of refrigerators has played a crucial role in improving nutrition and dietary habits (25). By revolutionizing food preservation, refrigerators have not only enhanced the nutritional value of food but also improved both its quality and availability (26, 27). Studies show that refrigeration technology is strongly linked to increased consumption of perishable foods, which positively impacts public health in developing countries (28, 29). However, the widespread use of refrigeration may also lead to dietary imbalances, increasing the risk of obesity and related health issues (30).

Despite the dual impact of refrigerator penetration on nutritional health, significant gaps remain in the literature regarding refrigeration technology, dietary structure, and health effects. Most studies have focused on developed markets, with limited research in developing countries. For example, Craig et al. (31) found that the widespread use of household refrigerators in the U.S. led to an eightfold increase in perishable food consumption. Research on developing countries typically targets specific groups or diseases. For instance, Karlsson and Subramanian (32) and Martinez et al. (33) examined the impact of refrigerator use on children’s nutrition, while Park et al. (34) explored the link between refrigerator use and gastric cancer mortality. These studies have limitations in sample selection and scope. Second, there is a “mechanism black box” issue. Heard et al. (35) found that refrigerators reduced staple food intake by 16% and increased meat consumption by 38% in Vietnam, but they did not clarify the specific pathways through which refrigeration technologies affect dietary outcomes. Finally, existing studies fail to address endogeneity issues, such as selectivity bias in refrigerator ownership, which can confound results and overestimate the net effect of refrigerators on dietary improvement. These methodological shortcomings undermine the causal validity of current findings, making it difficult to accurately assess the structural effects of refrigerator penetration on nutritional transition.

Using six waves of tracking data from the China Health and Nutrition Survey (CHNS), this study examines the dynamic effects of household refrigerator ownership on dietary structure and overweight/obesity risk. The goal is to provide policy guidance for developing countries facing health challenges during nutritional transition. The study approaches this in two dimensions: first, it analyzes changes in dietary structure by focusing on average daily calorie intake and the energy ratios of carbohydrates, fats, and proteins; second, it evaluates the health impacts by examining refrigerator ownership’s influence on overweight and obesity risk using BMI thresholds. To accurately identify these effects, the study employs the control function method to address endogeneity issues. The empirical results reveal the following: (1) Refrigerator ownership significantly increased daily calorie intake by 39.1%. Fat and protein energy ratios rose by 0.104 and 0.018 percentage points, respectively, while the carbohydrate energy ratio dropped by 0.12 percentage points, indicating a shift toward a higher energy-density diet; (2) The mediating mechanism tests identify two pathways: increased intake of high-fat and high-protein foods through larger purchases and increased consumption of processed foods; (3) Health effects exhibit significant heterogeneity. The risk of overweight increases with income, and the impact on older individuals is significantly higher than on younger groups. Additionally, males have more than double the obesity risk compared to females. Robustness tests, including variable substitution, sample size reduction, and urban–rural subgroup analysis, confirm the consistency of these findings.

This study makes academic innovations in three areas. Firstly, in terms of theory, it develops an analytical framework of “cold chain technology-nutritional transition-health effects,” highlighting the dynamic relationship between refrigerator ownership, dietary structure, and health risks. The study shows that refrigerators have a dual impact on the nutritional transition of Chinese residents: they improve dietary structure by increasing access to animal foods, but also raise the risk of overweight and obesity, emphasizing the complex health implications of technological advances. Secondly, on the methodological level, the study applies the control function method to resolve endogeneity issues in refrigerator usage decisions, enhancing the accuracy and reliability of the results. Thirdly, in terms of data application, it utilizes six waves of tracking data from the China Health and Nutrition Survey (CHNS, 1997–2011, *n* = 16,665), which covers a critical period for refrigerator adoption in China, offering unique data support. This research expands the intersection of behavioral economics and nutritional epidemiology, offering actionable policy insights for developing countries to overcome the “technological progress-health trap”.

## 2 Research background

With the rise in incomes among urban and rural residents and the implementation of China’s “Home Appliances to the Countryside” policy, the ownership of household refrigerators in China has surged from approximately 50 million units in 1997 to over 350 million units by 2024. Growth was rapid during the 1990s and early 2000s but has since slowed due to market saturation. Notably, the increase in refrigerator ownership among rural residents has been particularly remarkable. According to data from China’s home appliance industry and the China Statistical Yearbook, by 2023, refrigerator ownership exceeded 96%, with 103.4 refrigerators per 100 households. As lifestyles accelerate, younger consumers increasingly demand larger, smarter refrigerators to reduce purchase frequency and costs. Additionally, the rising living standards in rural areas have led to an increased reliance on purchased food, surpassing the consumption of self-produced goods. The expansion of cold-chain facilities further enhances the nutritional quality of residents’ diets (36). The COVID-19 pandemic has underscored the importance of refrigerators for food safety and nutritional health, heightening concerns about food storage and healthy eating.

**Figure 1:**
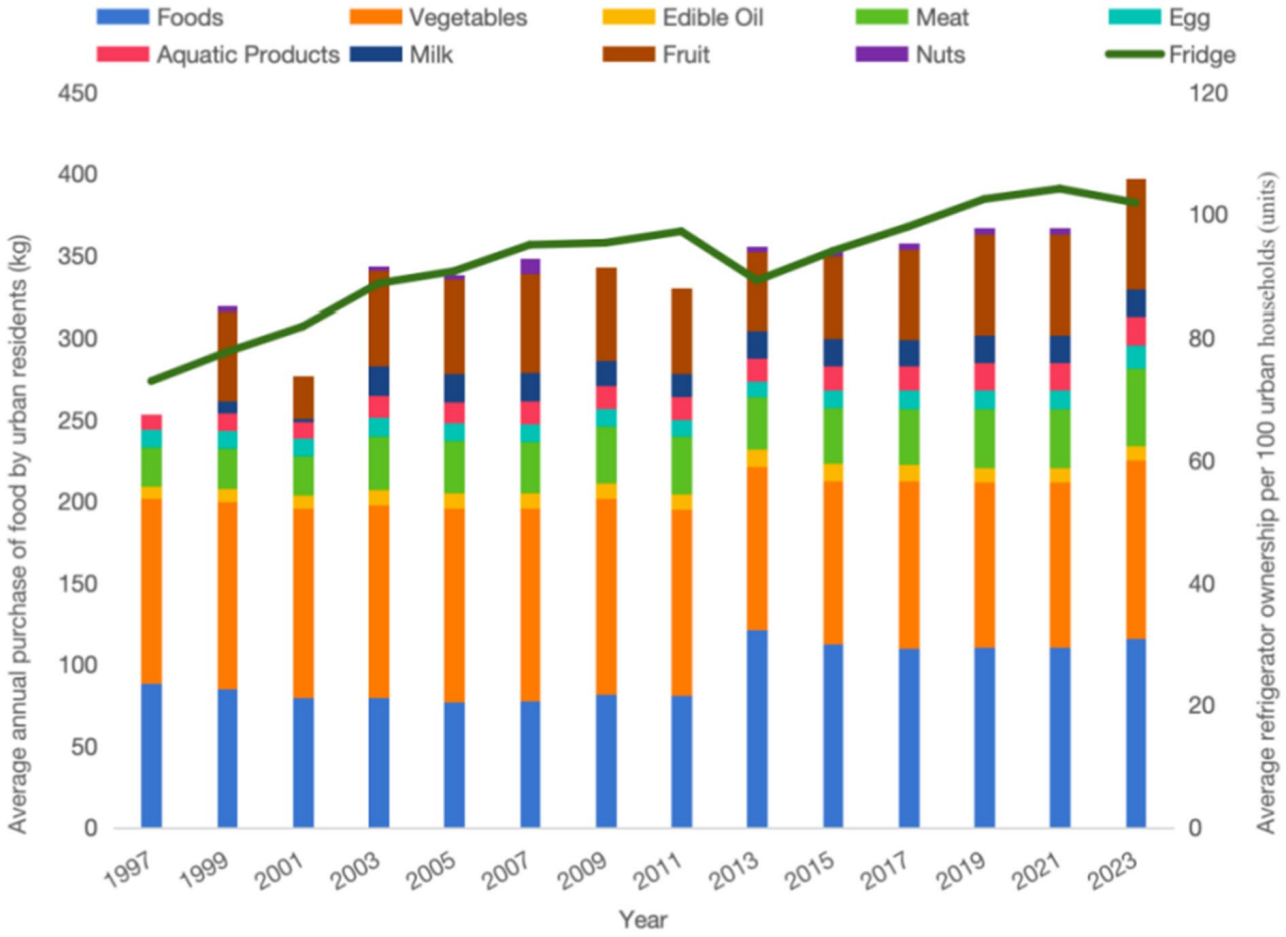
Graph of average annual food purchases per person and refrigerator ownership per 100 households in urban areas.

**Figure 2:**
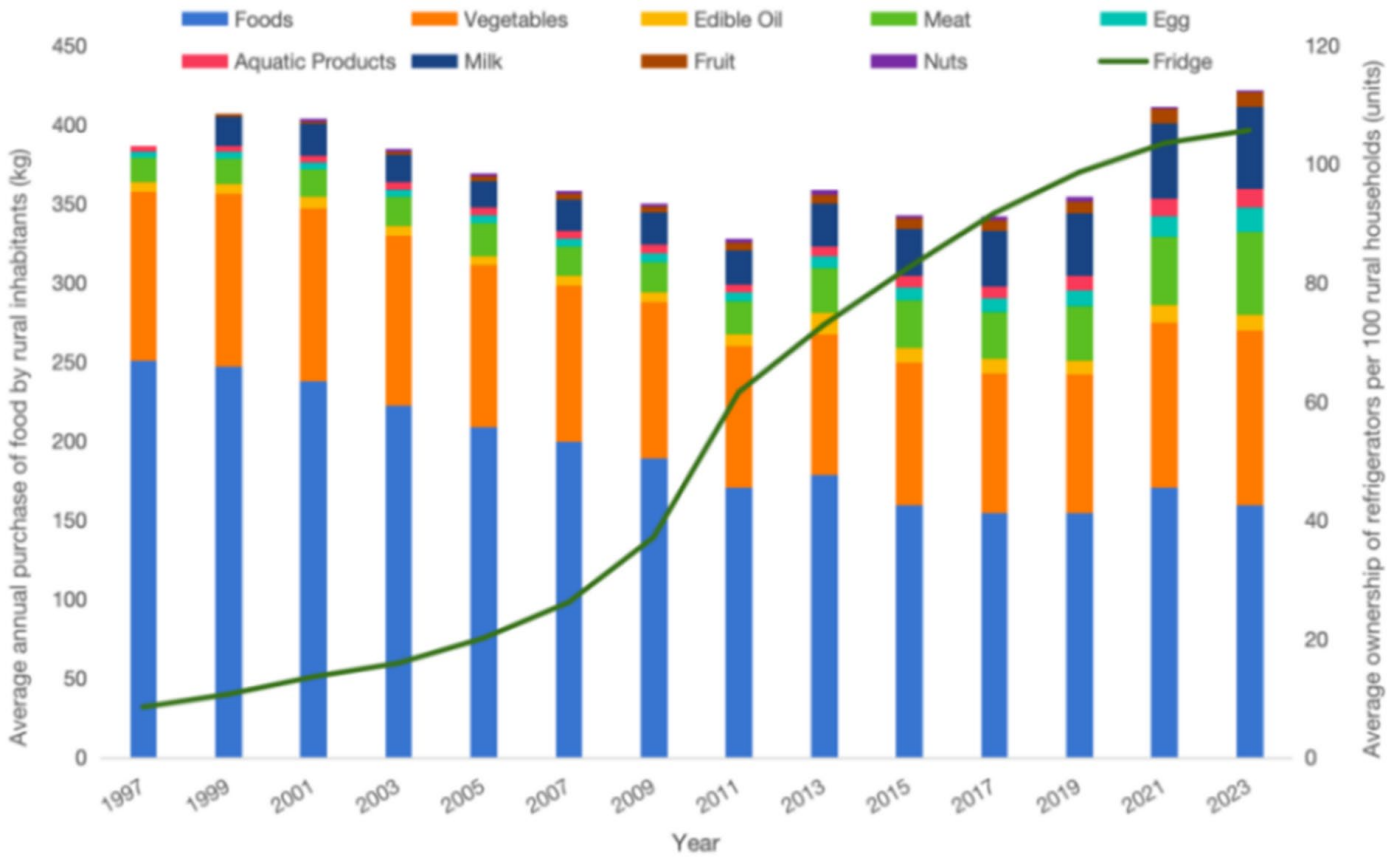
Graph of average annual food purchases per person and refrigerator ownership per 100 households in rural areas.

Figures 1, 2 illustrate the trends in per capita annual food consumption and refrigerator ownership per 100 households among urban and rural residents in China. As depicted in Figure 1, urban refrigerator ownership began at a high level, increasing from 72.98 units per 100 households in 1997 to 97.23 units per 100 households in 2011. Following a brief decline from 2011 to 2013, ownership resumed its upward trajectory, reaching 104.2 units per 100 households by 2021. In contrast, rural refrigerator ownership experienced rapid growth, rising from 8.49 units per 100 households in 1997 to 105.7 units per 100 households in 2023. From 1997 to 2011, per capita annual food consumption among urban residents decreased from 88.59 kg to 80.71 kg, with a brief increase in 2013 before resuming a downward trend. Meanwhile, per capita meat consumption increased steadily, and the proportion of aquatic products in total food consumption rose. Consumption of vegetables, fruits, and milk remained stable, while nut consumption was relatively low. In rural areas, the consumption of staple foods, particularly grains, has declined, while there has been a notable increase in the consumption of meat, eggs, aquatic products, milk, and fruits. Meat consumption, for example, grew from 15.08 kg in 1997 to 52.1 kg in 2023, a 245% increase. Vegetable consumption remained stable, and nut consumption remained low.

**Figure 3:**
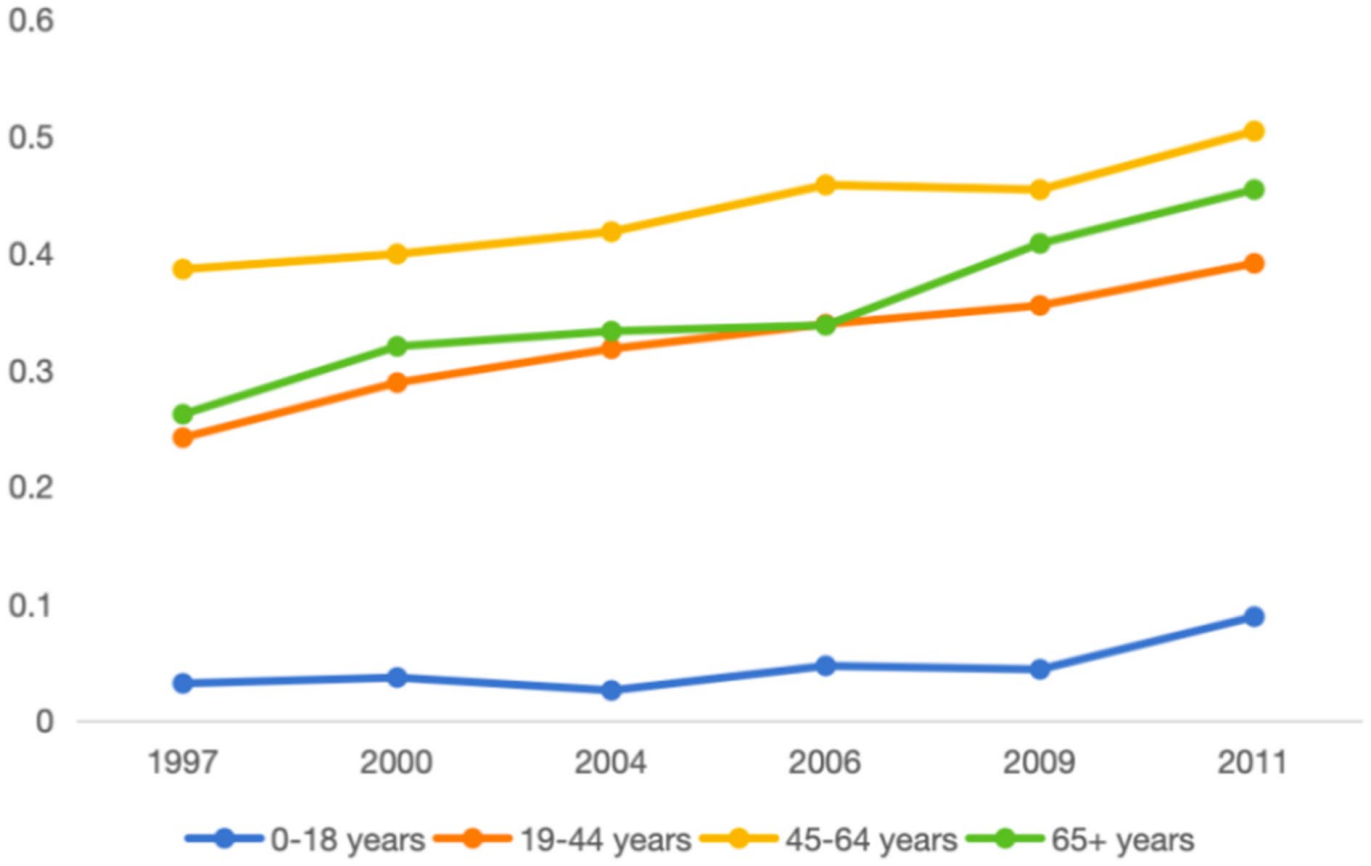
Overweight and obesity rates by four age groups.

Figure 3 illustrates the percentage of overweight and obese individuals across four age groups, based on sample data from the China Health and Nutrition Survey (CHNS) spanning from 1997 to 2011. The results indicate a clear upward trend in the rates of overweight and obesity among all age groups. The analysis suggests that the increase in refrigerator ownership, the upgrading of dietary structures, and the rising prevalence of overweight and obesity are significantly correlated trends. This observation raises the question of whether the increase in refrigerator ownership not only facilitates the improvement of dietary structures but also indirectly contributes to the worsening of overweight and obesity issues. This proposed causal relationship warrants empirical investigation for confirmation.

## 3 Data description

This study uses data from the six-wave China Health and Nutrition Survey (CHNS) conducted from 1997 to 2011. The CHNS, an international collaboration between the Carolina Population Center at the University of North Carolina at Chapel Hill and the National Institute of Nutrition and Health (NINH) of the Chinese Centers for Disease Control and Prevention (CCDC), covers nine provinces (Liaoning, Jiangsu, Shandong, Henan, Hubei, Hunan, Guangxi, Guizhou, and Heilongjiang) and three municipalities (Beijing, Chongqing, and Shanghai) in 2011. The data is crucial for analyzing the impact of China’s social and economic transformation on dietary and nutritional changes.

The survey used a multi-stage random cluster sampling method, with households selected from regions with varying economic development, public resources, and health indicators. It collected detailed data on household food consumption over three days, tracking both in-home and out-of-home meals for each household member. To focus on the impact of refrigerator ownership on food consumption and nutritional health, individuals who did not eat at home for the entire three days were excluded. Only those who ate exclusively at home during this period were retained. Additionally, samples with missing key explanatory variables were excluded, and continuous variables were Winsorized at the 1% level. The final dataset included 16,665 valid samples: 2,763 in 1997, 3,057 in 2000, 2,538 in 2004, 2,397 in 2006, 2,506 in 2009, and 3,404 in 2011. Following the approach of Tian and Lin (19) and Ren et al. (22), the study controlled for personal, family, and community characteristics to eliminate the influence of other factors on dietary structure, enhancing the reliability and validity of the findings.

### 3.1 Key variable description

This study uses two levels of data for the explanatory variables. First, dietary structure is measured by per capita daily calorie intake, and the calorie contribution ratios of carbohydrates, fats, and proteins. Second, body mass index (BMI) is used to assess overweight and obesity.

Caloric intake is a critical indicator of nutritional status in developing countries and carries significant implications for public health (37, 38). This study analyzes the nutritional intake characteristics of the sample population using data from the CHNS dietary survey. The results show that the average daily calorie intake was 2000.694 ± 760.168 kcal, with the macronutrient energy contribution ratios of carbohydrates, fat, and protein at 59.5, 27.4, and 12.6%, respectively (Table 1). These values were compared with the dietary energy supply standards recommended by the FAO, where fat and protein ratios are near the upper limit of the recommended range. To ensure comparability, energy conversion was performed using standardized factors: carbohydrate 4 kcal/g, fat 9 kcal/g, and protein 4 kcal/g. According to WHO and NHC guidelines, BMI values are classified as normal weight (18.5 kg/m^2^ ≤ BMI < 24.0 kg/m^2^), overweight (24.0 kg/m^2^ ≤ BMI < 28.0 kg/m^2^), and obesity (BMI ≥ 28.0 kg/m^2^).

**Table 1:** Descriptive statistics.

Variable	Variable description	Mean	Std. Dev.
Lnkcal	Logarithm of three-day average daily calorie intake	7.537	0.368
Carbohydrate	Ratio of carbohydrate energy contribution	0.595	0.124
Fat	Ratio of energy contribution from fat	0.274	0.118
Protein	Protein energy contribution ratio	0.126	0.029
Overweight	Overweight or not	0.287	0.452
Obese	Obese or not	0.089	0.285
Fridge	Dummy variable for whether the household owns a refrigerator	0.524	0.499
Kcal	Three-day average calorie intake	2000.694	760.168
Old-age ratio	Percentage of persons aged 60 and over in households	0.2	0.33
Children ratio	Percentage of children aged 14 and under in households	0.111	0.153
Age	Individual’s age	44.066	21.199
Lnhhincome	Per capita household income after adjusting for inflation	8.575	1.369
Supermarkets	Number of community supermarkets	1.992	7.595
Freemarkets	Number of community free markets	2.012	5.443
Bus	Number of community bus routes	0.664	0.472
Gender		1.557	0.497
Hhsize	Household size	3.603	1.484
Activity	individual activity intensity	2.706	1.425
Fridgenumber	Number of refrigerators in the household	0.544	0.566
Electrictyprice	Community cost per kilowatt-hour	0.705	1.367

The key explanatory variable in this study is a binary indicator of household refrigerator ownership. Households with a refrigerator are coded as 1, and those without as 0. Sample data show a steady increase in ownership, rising from 30.8% in 1997 to 66.6% in 2009, and reaching 84.4% by 2011. This long-term tracking study (1997–2011) is based on a large, nationally representative sample. The trend closely aligns with national data on refrigerator ownership per 100 households, confirming the sample’s strong representativeness.

### 3.2 Description of mechanism variables

The widespread use of refrigerators in modern households has significantly altered food purchasing and consumption behaviors. Research suggests that refrigerator use may contribute to overeating through two pathways, thereby increasing the risk of overweight and obesity. This paper examines the transmission mechanisms linking refrigerator use to overweight and obesity, drawing on insights from behavioral economics, nutrition, and environmental psychology.

#### 3.2.1 Refrigerator use leads to an increase in household food purchases

Refrigerators encourage increased purchasing behavior by enhancing food storage convenience. Their low-temperature preservation enhances food shelf life, minimizes spoilage risk, and reduces shopping time costs, thereby promoting a preference for bulk purchasing (30, 31). Behavioral studies show that when individuals perceive ample storage space, they tend to buy more, leading to a larger inventory that promotes the consumption of high-calorie convenience foods, increasing energy intake (39). This “storage space-purchase quantity” feedback loop results in a long-term saturation of household food inventory, creating “visible eating triggers”—increased food visibility and proximity, which encourage unplanned eating (40). Refrigerator capacity, combined with supermarkets’ marketing of large packaged products, also encourages higher purchases due to lower unit costs (41, 42). When large packages fill refrigerator space, consumers feel a psychological compulsion to “eat it all”, increasing daily intake. Urbanization has accelerated the pace of life, especially among younger people, raising the time cost of food shopping and prompting a “low-frequency, large-volume” purchasing strategy. Older individuals, shaped by previous material shortages, also tend to buy excess food. While refrigerators improve food preservation, their interaction with marketing strategies and psychological factors exacerbates over-purchasing and over-storage, contributing to the health risks of overweight and obesity.

#### 3.2.2 Refrigerator use leads to increased consumption of processed foods at home

The widespread popularity of household refrigerators symbolizes significant technological progress in food storage, facilitating the gradual integration of processed foods into daily diets through a “push-pull effect.” First, the optimization of cold chain systems and effective advertising have driven this trend. Advances in cold chain technology have extended the shelf life of pre-processed foods while reducing storage costs. Consequently, many food companies have leveraged the convenience of home refrigerators to introduce a variety of easy-to-store, ready-to-eat products, successfully capturing consumer interest. Moreover, marketing strategies for processed foods have further shaped this consumer trend. Second, shifts in consumer demands have also played a critical role. Urbanization has increased the demand for time-saving solutions, particularly among younger generations with declining cooking skills. This shift has made convenient processed foods more appealing to modern families. Processed foods are calorie-dense, often containing high levels of added sugars and fats, which contribute to weight gain (43). Furthermore, the combination of additives such as fat, salt, and sugar enhances flavor, which fosters consumer dependence and contributes to overconsumption. Increasing evidence suggests that the rising consumption of processed foods is a significant factor driving the prevalence of overweight and obesity (40, 44–46).

### 3.3 Description of control variables

Control variables were categorized into individual, household, and community levels (Table 1). At the individual level, food intake is influenced by social status, education, and personal characteristics. Therefore, we selected variables such as gender, age, and activity level (47, 48). At the household level, we included household size, the proportion of elderly individuals (aged 60 and above), and the proportion of children (under 14 years old) within the household size (38). At the community level, dietary consumption is affected by socio-economic factors, including the surrounding environment and food access. Thus, we considered the number of supermarkets within a 5 km radius, the number of free markets, and the presence of bus stops. These variables help control for the effects of community infrastructure and food availability on residents’ dietary behaviors (49).

## 4 Methods

### 4.1 Model design

Endogeneity may exist between household refrigerator ownership and dietary structure. Refrigerator ownership is influenced by both observable and unobservable factors. Unobservable factors, such as food culture and community demonstration effects, can influence household refrigerator ownership and dietary outcomes. Furthermore, since households may proactively purchase refrigerators to improve dietary health, bidirectional causality may exist between the explanatory variable and the outcome variable, thereby introducing endogeneity concerns. To address the estimation bias caused by endogeneity, this study employs the Control Function approach (CF approach) (50). As an instrumental variable for refrigerator ownership, we select community electricity prices (51). Refrigerators are high energy-consuming appliances, and their operating costs are significantly negatively correlated with community electricity prices. Lower electricity prices foster an environment conducive to refrigerator ownership. The first column of Table 2 indicates that electricity prices have a significantly negative effect on refrigerator ownership. Furthermore, the joint significance test of the F-statistic exceeds 10, suggesting a strong correlation between electricity prices and refrigerator ownership, with no issues related to weak instruments. Furthermore, community electricity prices, determined by government-guided pricing mechanisms, are based on electricity supply costs and are not directly related to nutrition and health, thus satisfying the exogenous assumption of the instrumental variable, Columns (2) and (3) of Table 2 confirm this view.

**Table 2:** Tests of instrumental variable assumptions.

Variables	(1)	(2)	(3)
	Fridge	Overweight	Obese
Electrictyprice	−0.010*** (0.003)	−0.003 (0.010)	0.012 (0.013)
Constant	−0.139*** (0.038)	−1.323 (0.125)	−2.244 (0.167)
Controls	Yes	Yes	Yes
*F*-value	245.81		
N	10,476	12,016	12,016

***, **, and * indicate significance at the 1, 5, and 10% levels, respectively. Robust standard errors are shown in parentheses.

The control function method introduces exogenous instrumental variables in the first stage to isolate the exogenous component of refrigerator ownership decisions. The residuals from the first-stage regression are then used as a new control variable in the original model, helping to account for unobserved factors correlated with the endogenous variables and correcting potential biases in the estimation results.

The specific model design is as follows:


(1) $$\;\text{Fridg}e_{\mathrm{it}}=\alpha_{0}+\alpha_{1}\text{Electricitypric}e_{\mathrm{ijt}}+\alpha_{2}X_{i}+\alpha_{3}Z_{j}+\in_{\mathrm{it}}$$

(2) $$\;Y_{\mathrm{it}}=\beta_{0}+\beta_{1}\text{Fridg}e_{\mathrm{it}}+\beta_{2}\hat{\in_{\mathrm{it}}}+\beta_{3}X_{i}+\beta_{4}Z_{j}+\mu_{\mathrm{it}}$$

(3) $$P({\text{Overweight}}_{i}/{\text{obese}}_{i}=1)=\Phi (\theta_{0}+\theta_{1}\text{Fridg}e_{\mathrm{it}}+\theta_{2}X_{i}+\theta_{3}Z_{j})$$

In Equation 1, let $\text{Fridg}e_{\mathrm{it}}$ be a dummy variable indicating whether the household of individual i owns a refrigerator in year t (1 = owns, 0 = does not own); $\text{Electricitypric}e_{\mathrm{ijt}}$ represent the electricity price in the community where the individual resides in year t (instrumental variable); let $X_{i}$ be the characteristic variables affecting individual dietary structure; let $Z_{j}$ denote the characteristic variables of the community. In Equation 2, let $Y_{\mathrm{it}}$ represent the daily calorie intake and the energy contribution ratios of three macronutrients; $\epsilon_{\mathrm{it}}$ , $\mu_{\mathrm{it}}\;$ be the random error terms; and let ${\hat{\epsilon }}_{\mathrm{it}}$ be the residual from the first-stage regression. For binary variables (e.g., overweight and obesity status), we use Equation 3.

To further elucidate the pathways through which refrigerator ownership influences overweight and obesity, a three-stage mediation model was employed to assess the mediating effects of food purchases and processed food consumption on these outcomes. Given that Equation 3 has already addressed the overall impact of refrigerator ownership on overweight and obesity, Equation 4 is formulated to investigate the effect of refrigerator ownership on the aforementioned pathways. Additionally, Equation 5 integrates both refrigerator ownership and the identified mediating variables in the model for a joint significance test.

The specific econometric models are as follows:


(4) $$M_{\mathrm{it}}=\eta_{0}+\eta_{1}\text{Fridg}e_{\mathrm{it}}+\eta_{2}X_{i}+\eta_{3}Z_{j}+\varphi_{\mathrm{it}}$$

(5) $$\begin{array}{l} \;{\text{Overweight}}_{i}/{\text{obese}}_{i}=\gamma_{0}+\gamma_{1}M_{\mathrm{it}}+\gamma_{2}\text{Fridg}e_{\mathrm{it}}\\ +\gamma_{3}X_{i}+\gamma_{4}Z_{j}+\omega_{\mathrm{it}} \end{array}$$

$\;M_{\mathrm{it}}$ represent the amount of food purchased and the quantity of processed foods consumed. The remaining variables are as above.

### 4.2 Analysis of regression results

To mitigate estimation bias stemming from self-selection and endogeneity, daily calorie intake was estimated using the least squares method. The ratios of energy contributions from carbohydrates, fats, and proteins were estimated with a Tobit model. Given that overweight and obesity are binary variables, they were analyzed using a Probit model. The empirical results of the benchmark regression are presented in Table 3 and Supplementary Table 1.

**Table 3:** Regression results of refrigerator ownership on dietary composition and overweight/obesity.

Variables	(1)	(2)	(3)	(4)	(5)	(6)
	Lnkcal	Carbohydrate	Fat	Protein	Overweight	Obese
Fridge	0.391^***^ (0.033)	−0.120^***^ (0.011)	0.104^***^ (0.011)	0.018^***^ (0.003)	0.194^***^ (0.029)	0.259^***^ (0.043)
Residuals	−0.410^***^ (0.033)	0.0606^***^ (0.011)	−0.0516^***^ (0.011)	−0.0111^***^ (0.003)		
Controls	Yes	Yes	Yes	Yes	Yes	Yes
Constant	7.751^***^ (0.033)	0.651^***^ (0.010)	0.236^***^ (0.010)	0.108^***^ (0.002)	−1.327^***^ (0.127)	−2.279^***^ (0.181)
*N*	10,145	10,145	10,145	10,145	10,558	10,558

***, **, and * indicate significance at the 1, 5, and 10% levels, respectively. Robust standard errors are shown in parentheses. The residuals from the first stage of the overweight and obesity variables are not significant, which proves that refrigerator ownership does not have endogeneity with respect to overweight and obesity, indicating that the Probit model results are unbiased. Therefore, only the results of the Probit model are presented in the fifth and sixth columns.

As shown in Table 3 and Supplementary Table 1, the residuals from the first stage are all significant in the second stage, indicating an endogeneity problem with refrigerator ownership. This suggests that some endogeneity bias can be corrected using the control function method. One regression result in column (1) reveals that a 1% increase in household refrigerator ownership leads to a significant 39.1% increase in an individual’s average daily calorie intake (*p* < 0.01). Further analysis of nutrient energy ratios in columns (2)–(4) indicates that refrigerator use has a significantly negative marginal effect on the carbohydrate energy ratio (*β* = −0.120), while it positively affects the fat and protein energy ratios (β = 0.104 and β = 0.018, respectively), with the fat energy ratio being significantly higher than that of protein. The empirical results confirm that the widespread adoption of refrigerators has significantly driven the shift in dietary patterns from a “carbohydrate-dominant” diet to one that is “lipoprotein-enriched.” This finding provides micro-evidence for understanding the technology-driven pathways of the nutrition transition.

The potential impact of household refrigerator ownership on obesity and overweight is further examined. The probit regression results in column (5) of Table 3 indicate that owning a refrigerator significantly increases the probability of being overweight, with a marginal effect of 0.194 (*p* < 0.01). A more detailed analysis in column (6) shows that refrigerator ownership has a marginal effect of 0.259 (*p* < 0.01) on obesity. Refrigerators drive systematic changes in energy intake and metabolic pathways, promoting high-fat, high-protein dietary patterns. Specifically, the increased fat and protein energy ratios raise calorie density, ultimately heightening the risk of obesity and overweight (52, 53). These findings underscore the intrinsic link between household technological innovations and the prevalence of nutritional diseases, providing a solid evidence base for public health policy formulation.

### 4.3 Analysis of the results of the mediation effect

The empirical results presented in Table 4 elucidate the mediating mechanism underlying the health impacts of refrigerator ownership. The findings from the second step of the mediation effect test, as shown in columns (1) and (4), indicate that refrigerator ownership significantly increases households’ total food purchases and consumption of processed foods. Further results from the third step of the mediation effects test, detailed in columns (2)–(3) and (5)–(6), suggest that both food purchases and processed food consumption significantly mediate the relationship between refrigerator ownership and overweight/obesity. Specifically, the joint significance test in columns (2)–(3) confirms that refrigerator ownership indirectly contributes to the rise in overweight and obesity prevalence by increasing the quantity of food purchased. Similarly, the results in columns (5)–(6) demonstrate that the rise in processed food consumption is also a crucial pathway through which refrigerator ownership influences the prevalence of overweight and obesity. This finding aligns with the conclusions of Neven et al. (54) regarding the impact of refrigeration technology on food consumption patterns, affirming that refrigerator ownership exacerbates overweight and obesity health issues by prolonging food freshness, altering the purchasing patterns of single-person households, and increasing the volume of processed foods purchased.

**Table 4:** Mediation analysis results.

Variables	(1)	(2)	(3)	(4)	(5)	(6)
	Food purchased	Overweight	Obese	Processed foods consumed	Overweight	Obese
Fridge	0.117*** (0.037)	0.195*** (0.029)	0.267*** (0.040)	0.077*** (0.013)	0.193*** (0.029)	0.264*** (0.040)
Food purchased		0.015** (0.007)	0.022** (0.010)			
Processed foods consumed					0.041** (0.018)	0.051** (0.022)
Controls	Yes	Yes	Yes	Yes	Yes	Yes
Constant	3.425*** (0.153)	−1.358*** (0.127)	−2.315*** (0.169)	−0.190*** (0.056)	−1.292*** (0.124)	−2.219*** (0.164)
*N*	12,104	10,558	10,558	12,104	10,558	10,558

***, ** and * indicate significant at the 1, 5 and 10% levels, respectively, standard errors are in parentheses and CVs are control variables.

## 5 Robustness analysis

### 5.1 Replacing key explanatory variables

This study performed a robustness test utilizing a variable substitution method, converting refrigerator ownership from a binary dummy variable to a continuous quantitative indicator. The regression results presented in Supplementary Table 2 indicate that the energy contribution of carbohydrates to the refrigerator is negatively correlated (*β* = −0.050, *p* < 0.01). In contrast, it increases the energy share from fat (β = 0.044, *p* < 0.01) and protein (β = 0.006, *p* < 0.01). Regarding health effects, Probit model estimation indicates a significant positive marginal effect of refrigerator ownership on the probability of being overweight and obese. Specifically, an increase in the number of refrigerators enhances individuals’ fat and protein energy contributions by improving the storage capacity for animal foods. This finding aligns with the regression results obtained from the control function method, confirming the robustness of the study’s conclusions.

### 5.2 Narrowing the sample range

In this study, a robustness test was conducted using a sample screening method based on the design of Ren et al. (15). This approach limited the sample to the working-age population, specifically individuals aged 18 to 65, to eliminate the influence of distinct dietary patterns among children, adolescents, and seniors. The estimation results in Supplementary Table 2 reveal that, after controlling for individual heterogeneity, refrigerator ownership significantly reduces the carbohydrate energy share (*β* = −0.053). Simultaneously, it increases the marginal elasticity of fat intake (β = 0.047, *p* < 0.01) and protein intake (β = 0.006, p < 0.01). Regarding health effects, the results in columns (6) and (7) of Supplementary Table 2 show that household refrigerator ownership positively influences the likelihood of overweight and obesity. These results remain consistent with the benchmark regression.

### 5.3 Separate urban and rural samples

This study employs a spatial heterogeneity analysis framework to account for systematic differences between urban and rural areas, focusing on economic development, infrastructure density, and food supply chain maturity through a hierarchical regression model. As shown in Supplementary Table 2, the urban–rural sub-sample regression reveals significant policy targeting effects. In terms of nutritional structure, both urban and rural residents exhibit a “carbohydrate substitution” effect. For urban residents, refrigerator ownership reduces the proportion of carbohydrate energy (*β* = −0.047), while for rural residents, the decrease is even greater (β = −0.054). The marginal elasticity of fat and protein intake is significantly higher in the rural sample. Furthermore, refrigerator ownership among urban residents positively influences the probability of being overweight at the 10% significance level. Notably, the elasticity coefficients for overweight and obesity risk associated with refrigerator ownership among rural residents are 0.193 and 0.303 (*p* < 0.01), respectively, exceeding those observed in the urban sample. This finding supports the hypothesis of “technology shock amplification” in regions with underdeveloped infrastructure—when the diffusion of refrigeration technology outpaces local advancements in dietary knowledge, it exacerbates the accumulation of health risks.

## 6 Heterogeneity analysis

### 6.1 By income

Household income significantly influences the dietary consumption structure of the population. This study categorizes sample households based on three income quartiles: those below the first quartile (25th percentile) are classified as the low-income group, those between the 25th and 75th percentiles as the middle-income group, and those above the third quartile (75th percentile) as the high-income group. The empirical results in Supplementary Table 3 show that refrigerators, as key household capital goods, have a pronounced income gradient effect on nutritional improvement. For low-income households, refrigerators significantly increase the energy contribution ratios of fat (*β* = 0.056) and protein (β = 0.009), indicating that access to this consumer durable enhances the nutritional quality of their diets. Notably, the risk of overnutrition rises with income level. In the low-income group, the effect of refrigerators on overweight and obesity is significantly positive at the 5% level, while the effect on overweight in middle-and high-income groups significantly positive at the 1% level. Refrigerators significantly raise the probability of obesity in the middle-income group but have no impact in the high-income group. This finding aligns with Malik et al. (55), which suggests that affluent households, having the means to purchase healthy foods, the time for physical activity, and access to quality healthcare, experience lower obesity rates. These results reveal a non-linear relationship between household income and nutritional transition: the welfare effects of advancements in food storage technology may diminish beyond a certain income threshold. This insight supports the need for precision nutritional intervention policies.

### 6.2 By age

This study employs the life cycle consumption theory to develop an age heterogeneity analysis framework. The sample is divided into four groups: young (≤35 years), middle-aged (36–49 years), prime-aged (50–64 years), and old (≥65 years). After accounting for generational differences, significant life cycle characteristics of refrigerator usage on nutrition were identified. As shown in Supplementary Table 4, the elderly group exhibited the largest decrease in carbohydrate energy share (*β* = 0.077, *p* < 0.01). The fat intake share coefficient was 0.071 (*p* < 0.01), notably higher than in other groups. Regarding health impacts, refrigerator ownership significantly correlated with an increase in overweight individuals in the elderly group (β = 0.294, *p* < 0.01). This phenomenon is linked to the decline in basal metabolic rate among the elderly and reduced energy consumption. The refrigerator’s storage capability fosters a metabolic imbalance characterized by “low energy consumption-high intake.” Additionally, refrigerator ownership influenced obesity across all groups, with the senior age group experiencing the most pronounced effect (β = 0.259, *p* < 0.01).

### 6.3 By gender

This study employed a sub-sex regression model to examine the heterogeneity in nutritional effects resulting from refrigerator ownership. The empirical findings presented in Supplementary Table 5 reveal notable gender differences. Refrigerators significantly increased the fat-energy share in men more than in women. Regarding health risks, the Probit model indicates that each unit increase in refrigerator ownership doubles the impact on overweight and obesity risk for men compared to women.

## 7 Conclusions and policy implications

Using tracking data from the China Health and Nutrition Survey (CHNS), this study systematically examines the dynamic effects of household refrigerator ownership on dietary structure and health risks. It focuses on changes in average daily calorie intake and the energy ratios of the three major nutrients, assessing the risk of overweight and obesity based on BMI standards. To correct for selective and endogenous bias, the study employs the control function method. The findings reveal that: first, refrigerator ownership significantly alters the dietary energy structure. This is evident in the increased energy supply ratios of fat and protein, coupled with a decrease in carbohydrate energy supply. Second, the popularity of refrigerators drives increased food purchases and the integration of processed foods into diets, leading to higher dietary fat accumulation. Moreover, the health effects exhibit significant group heterogeneity. There is a positive correlation between income levels and the risk of being overweight; older individuals exhibit a greater sensitivity to this risk compared to the younger counterparts, and men have a higher likelihood of being overweight or obese than women. The findings are robust across multiple dimensions. These insights hold substantial theoretical and practical value in balancing technological advancements with public health objectives.

During the nutritional transition in developing countries, it is vital to guide the impact of universal refrigerator access on dietary patterns. This study offers several systematic policy recommendations.

First, build a synergistic system of health-oriented cold chain facilities and nutritional interventions. Incorporating the construction of cold-chain facilities into the indicators for assessing healthy cities is essential, along with strengthening policy linkages with public health. Simultaneously, targeted nutritional health education should be implemented through diversified channels, such as social media and community outreach. Special attention must be given to precise interventions for populations at higher dietary risk, including the elderly, men, and middle-income and high-income groups.

Second, a nuanced intervention strategy should be developed to scientifically guide the population’s food purchasing behavior. Precise nutritional intervention strategies can be tailored to different population characteristics. For the time-sensitive youth demographic, an intelligent procurement decision support system can be developed that utilizes big data to analyze their consumption habits and provide personalized procurement recommendations, thereby reducing irrational hoarding behaviors. For the cognitively established elderly population, intuitive methods such as visual nutrition loss experiments and food spoilage case comparisons can be employed to address their tendency to over store. Through a tiered approach, we aim to achieve a balanced development of health and convenience.

Third, improving the nutritional regulation and health guidance policy system for processed foods. The risk warning labeling system has been strengthened. Building on the existing nutrition labeling standards, a graded warning system has been implemented for processed foods high in sugar, fat, salt, and trans fatty acids, utilizing red labels and other conspicuous methods to alert consumers to health risks and enhance their awareness.

This study empirically analyzes the impact of refrigerator ownership on dietary structure and nutritional health. It is important to note that due to data timeliness limitations, the findings have limitations in reflecting current trends in food consumption. Future research will focus on the interactions between online shopping, refrigerator storage, and dietary health. Regarding nutritional health indicators, this study employs the BMI index, drawing from existing mainstream literature to measure health outcomes such as overweight and obesity. However, significant differences exist in the effects of various protein and fat sources on weight gain (56). Future research should address this gap by conducting a more detailed analysis of food composition to elucidate the mechanisms. In terms of data collection methods, the three-day food recall method employed in this study, although widely used, may still be prone to measurement errors. To enhance the robustness of the findings, future studies should incorporate field research and more rigorous food recording methods (e.g., weighing methods, dietary diaries, etc.) to obtain more accurate food consumption data, thereby providing a more reliable empirical foundation for related research.

## Data Availability

The raw data supporting the conclusions of this article will be made available by the authors, without undue reservation.
