# Supplementary material for: Refrigerator ownership and the nutrition-health trade-off: evidence from Chinese households
Source: Front Nutr. 2025 Jul 8;12:1620134. doi: 10.3389/fnut.2025.1620134 (PMC12279700; doi:10.3389/fnut.2025.1620134)
Supplement: Supplementary file 1 [file Table_1.docx]

Supplementary material

Table 1 Regression results of refrigerator ownership on dietary composition and overweight/obesity

|  | (1) | (2) | (3) | (4) | (5) | (6) |
| --- | --- | --- | --- | --- | --- | --- |
|  | Lnkcal | Carbohydrate | Fat | Protein | Overweight | Obese |
| Fridge | 0.391^***^ | -0.120^***^ | 0.104^***^ | 0.0182^***^ | 0.194^***^ | 0.259^***^ |
|  | (0.033) | (0.011) | (0.011) | (0.003) | (0.029) | (0.043) |
| Residuals | -0.410^***^ | 0.0606^***^ | -0.0516^***^ | -0.0111^***^ |  |  |
|  | (0.033) | (0.011) | (0.011) | (0.003) |  |  |
| Gender | -0.134^***^ | 0.00453^*^ | 0.00494^*^ | 0.000804 | -0.035 | 0.130*** |
|  | (0.007) | (0.002) | (0.002) | (0.001) | (0.027) | (0.039) |
| Age | 0.000856^***^ | -0.000251^***^ | 0.000136^*^ | -0.0000402^*^ | 0.016*** | 0.012*** |
|  | (0.000) | (0.000) | (0.000) | (0.000) | (0.001) | (0.001) |
| Hhsize | -0.0147^***^ | 0.0132^***^ | -0.0144^***^ | 0.00159^***^ | -0.008 | 0.018 |
|  | (0.003) | (0.001) | (0.001) | (0.000) | (0.010) | (0.015) |
| Supermarkets | 0.000102 | -0.000688^***^ | 0.000513^**^ | 0.000154^**^ | 0.002 | -0.002 |
|  | (0.001) | (0.000) | (0.000) | (0.000) | (0.003) | (0.004) |
| Freemarkets | -0.00555^***^ | 0.000369 | -0.000367 | 0.0000197 | 0.000 | 0.005 |
|  | (0.001) | (0.000) | (0.000) | (0.000) | (0.003) | (0.005) |
| Old-age ratio | -0.123^***^ | -0.0165^***^ | 0.0126^**^ | 0.00688^***^ | -0.469*** | -0.236*** |
|  | (0.013) | (0.004) | (0.004) | (0.001) | (0.050) | (0.069) |
| Children ratio | -0.0594^*^ | -0.0133 | 0.0160^*^ | -0.00242 | -0.535*** | -0.365** |
|  | (0.025) | (0.008) | (0.008) | (0.002) | (0.110) | (0.164) |
| Activity | 0.0577^***^ | 0.00665^***^ | -0.00544^***^ | -0.00129^***^ | -0.022** | -0.001 |
|  | (0.004) | (0.001) | (0.001) | (0.000) | (0.011) | (0.015) |
| Lnhhincome | -0.0245^***^ | -0.00400^**^ | 0.00236 | 0.000628 | 0.022** | -0.000 |
|  | (0.004) | (0.001) | (0.001) | (0.000) | (0.011) | (0.015) |
| Bus | -0.0798^***^ | -0.0181^***^ | 0.0163^***^ | -0.00239^***^ | 0.027 | 0.062 |
|  | (0.008) | (0.002) | (0.002) | (0.001) | (0.029) | (0.041) |
| constant | 7.751^***^ | 0.651^***^ | 0.236^***^ | 0.108^***^ | -1.327*** | -2.279*** |
|  | (0.033) | (0.010) | (0.010) | (0.002) | (0.127) | (0.181) |
| *N* | 10145 | 10145 | 10145 | 10145 | 10558 | 10558 |

Note: ***, **, and * indicate significance at the 1%, 5%, and 10% levels, respectively. Robust standard errors are shown in parentheses.

Table 2 Robustness Test

| Replace Key Explanatory Variables | | | | | | |
| --- | --- | --- | --- | --- | --- | --- |
| Variable | (1) | (2) | (3) | (5) | (6) | (7) |
|  | Lnkcal | Carbohydrate | Fat | Protein | Overweight | Obese |
| Fridgenumber | -0.023*** | -0.050*** | 0.044*** | 0.006*** | 0.158*** | 0.227*** |
|  | （0.006） | (0.002) | (0.002) | (0.001) | (0.028) | (0.035) |
| *N* | 10217 | 10217 | 10217 | 10217 | 9581 | 9581 |
| Narrow the Sample Range | | | | | | |
| Fridge | 0.021 | -0.053*** | 0.047*** | 0.006*** | 0.155*** | 0.257*** |
|  | (0.027) | (0.003) | (0.003) | (0.001) | (0.037) | (0.049) |
| *N* | 7692 | 7090 | 7090 | 7088 | 6688 | 6688 |
| Urban Resident Sample | | | | | | |
| Fridge | 0.111* | -0.047*** | 0.043*** | 0.005*** | 0.163* | 0.066 |
|  | (0.055) | (0.005) | (0.005) | (0.002) | (0.067) | (0.088) |
| *N* | 2891 | 2679 | 2679 | 2679 | 2505 | 2505 |
| Rural Resident Sample | | | | | | |
| Fridge | 0.006 | -0.054*** | 0.048*** | 0.006*** | 0.193*** | 0.303*** |
|  | (0.028) | (0.003) | (0.003) | (0.001) | (0.037) | (0.050) |
| *N* | 8208 | 7538 | 7538 | 7538 | 7076 | 7076 |

Note: ***, ** and * indicate significant at the 1%, 5% and 10% levels, respectively, standard errors are in parentheses.

Table 3 Heterogeneity analysis (1)

| Low-income families | | | | | | |
| --- | --- | --- | --- | --- | --- | --- |
| Variable | (1) | (5) | (6) | (7) | (8) | (9) |
|  | Lnd3kcal | Carbohydrate | Fat | Protein | Overweight | Obese |
| Fridge | -0.047** | -0.065*** | 0.056*** | 0.009*** | 0.180** | 0.290** |
|  | (0.017) | (0.005) | (0.005) | (0.001) | (0.065) | (0.089) |
| N | 2937 | 2937 | 2937 | 2937 | 2642 | 2642 |
| Middle-income households | | | | | | |
| Fridge | -0.013 | -0.059** | 0.052* | 0.006*** | 0.162*** | 0.265*** |
|  | (0.009) | (0.003) | (0.003) | (0.001) | (0.040) | (0.054) |
| N | 5940 | 5940 | 5940 | 5940 | 5553 | 5553 |
| High-income households | | | | | | |
| Fridge | -0.026 | -0.035*** | 0.036*** | 0.006*** | 0.301*** | 0.188 |
|  | (0.018) | (0.006) | (0.006) | (0.002) | (0.075) | (0.100) |
| N | 2449 | 2449 | 2449 | 2449 | 2363 | 2363 |

Note: ***, ** and * indicate significant at the 1%, 5% and 10% levels, respectively, standard errors are in parentheses.

Table 4 Heterogeneity analysis (2)

| Youth group | | | | | | |
| --- | --- | --- | --- | --- | --- | --- |
| Variable | (1) | (5) | (6) | (7) | (8) | (9) |
|  | Lnd3kcal | Carbohydrate | Fat | Protein | Overweight | Obese |
| Fridge | -0.073** | -0.067*** | 0.056*** | 0.010*** | 0.111 | 0.323** |
|  | (0.012) | (0.004) | (0.004) | (0.001) | (0.064) | (0.099) |
| N | 3862 | 3862 | 3862 | 3862 | 3442 | 3442 |
| Middle-age group | | | | | | |
| Fridge | -0.013 | -0.052*** | 0.049*** | 0.005*** | 0.168** | 0.233** |
|  | (0.013) | (0.004) | (0.004) | (0.001) | (0.057) | (0.760) |
| N | 2574 | 2574 | 2574 | 2574 | 2446 | 2446 |
| Senior group | | | | | | |
| Fridge | -0.006 | -0.056*** | 0.048*** | 0.006*** | 0.155** | 0.259*** |
|  | (0.013) | (0.005) | (0.005) | (0.001) | (0.056) | (0.073) |
| N | 2859 | 2859 | 2859 | 2859 | 2752 | 2752 |
| Elderly groups | | | | | | |
| Fridge | 0.033 | -0.077*** | 0.071*** | 0.009*** | 0.294*** | 0.228* |
|  | (0.017) | (0.006) | (0.006) | (0.002) | (0.072) | (0.093) |
| N | 2031 | 2031 | 2031 | 2031 | 1918 | 1918 |

Note: ***, ** and * indicate significant at the 1%, 5% and 10% levels, respectively, standard errors are in parentheses.

Table 5 Heterogeneity analysis (3)

| Male | | | | | | |
| --- | --- | --- | --- | --- | --- | --- |
| Variable | (1) | (2) | (3) | (4) | (5) | (6) |
|  | Lnd3kcal | Carbohydrate | Fat | Protein | Overweight | Obese |
| Fridge | -0.026* | -0.065*** | 0.059*** | 0.008*** | 0.269*** | 0.419*** |
|  | (0.004) | (0.003) | (0.003) | (0.001) | (0.045) | (0.066) |
| N | 5020 | 5020 | 5020 | 5020 | 4638 | 4638 |
| Female | | | | | | |
| Fridge | -0.025** | -0.063*** | 0.055*** | 0.009*** | 0.135*** | 0.172*** |
|  | (0.010) | (0.003) | (0.003) | (0.001) | (0.039) | (0.051) |
| N | 6306 | 6306 | 6306 | 6306 | 5920 | 5920 |

Note: ***, ** and * indicate significant at the 1%, 5% and 10% levels, respectively, standard errors are in parentheses.
